# Species diversity and drivers of arbuscular mycorrhizal fungal communities in a semi-arid mountain in China

**DOI:** 10.7717/peerj.4155

**Published:** 2017-12-08

**Authors:** He Zhao, Xuanzhen Li, Zhiming Zhang, Yong Zhao, Jiantao Yang, Yiwei Zhu

**Affiliations:** College of Forestry, Henan Agricultural University, Zhengzhou, China

**Keywords:** Illumina sequencing, AMF communities, Semi-arid field, Soil properties

## Abstract

Arbuscular mycorrhizal fungi (AMF) play an essential role in complex ecosystems. However, the species diversity and composition of AMF communities remain unclear in semi-arid mountains. Further, it is not well understood if the characteristics of AMF community assemblies differ for different habitat types, e.g., agricultural arable land, artificial forest land, natural grassland, and bush/wood land. Here, using the high-throughput technology by Illumina sequencing on the MiSeq platform, we explored the species diversity and composition of soil AMF communities among different habitat types in a semi-arid mountain (Taihang Mountain, Mid-western region of China). Then, we analyzed the effect of nutrient composition and soil texture on AMF community assembly. Our results showed that members of the *Glomus* genera were predominated in all soil types. The distance-based redundancy analysis indicated that the content of water, available phosphorus, and available potassium were the most crucial geochemical factors that significantly affected AMF communities (*p* < 0.05). The analysis of the soil texture confirmed that AMF diversity was negatively correlated with soil clay content. The comparison of AMF diversity among the various habitat types revealed that the artificial forest land had the lowest AMF diversity in comparison with other land types. Our findings suggest that there were differences in species diversity and composition of soil AMF communities among different habitat types. These findings shed new light on the characteristics of community structure and drivers of community assembly in AMF in semi-arid mountains, and point to the potential importance of different habitat types on AMF communities.

## Introduction

Arbuscular mycorrhizal fungi (AMF) play a high-value role for ecosystem restoration and sustainability (Herder et al., 2010; Sanders, 2010; Verbruggen et al., 2012). The majority of land plant species have the potential ability to form symbiotic relationships with AMF, which can significantly enhance plant growth (Lekberg & Koide, 2005), improve soil structure (Piotrowski et al., 2004; Caravaca et al., 2006; Wilson et al., 2009), and contribute to plant resistance to environmental stress (Benjamina, Karl & Johnn, 2009; Balliu, Sallaku & Rewald, 2015). AMF also can maintain ecosystems stability and promote ecosystem development (Larsen, Williams & Kremen, 2005; Fuhrman, 2009; Rosindell, Hubbell & Etienne, 2011). Therefore, to explore the ecological environment in diverse regions, understanding AMF diversity and biogeography will be of primary importance (Fitter, 2005; Chaudhry et al., 2012).

In recent years, many studies have reported the AMF community composition in different environmental conditions (Öpik et al., 2006; Wubet et al., 2006; Heijden & Scheublin, 2007; Lee, Lee & Young, 2008; Krüger et al., 2009). Scholars have argued that the composition of AMF communities will vary along the gradients of land-use intensity under the same climatic conditions and region of agricultural ecosystems (Dumbrell et al., 2010; Fritz et al., 2010; Lekberg et al., 2011; Mirás-Avalos et al., 2011; Meadow & Zabinski, 2012). Also, several papers have confirmed that the AMF distributions are caused by their ability to tolerate high nutrient concentrations in different habitat types (Porras-Alfaro et al., 2007; Egertonwarburton, Johnson & Allen, 2008; Thomson, Robson & Abbott, 2010). Meanwhile, through the investigation of natural or agricultural habitats, scholars have shown that a high diversity of rhizosphere AMF was found in natural habitat (Öpik et al., 2008; Bonfim et al., 2016), and the AMF communities inhabiting plant roots tended to have a lower diversity in agricultural ecosystems (Daniell et al., 2001; Alguacil et al., 2011; Schnoor et al., 2011; Bainard et al., 2015). However, most of the previous research works focused on single ecosystems (Helgason et al., 1998; Lumini et al., 2010; Verbruggen & Toby, 2010), and there are no comparative analyses on the AMF condition among different soil types under the same climate conditions in semi-arid regions.

Hitherto, traditional studies of AMF community composition have been scarce, partly due to the limitations of spore morphological features, which are easily influenced by external disturbances (Oehl et al., 2004), such as integrity of the spores (e.g., ability to identify spores). Due to the above defects, new research technologies are constantly updated. For instance, the development of molecular methods has greatly facilitated the studies of AMF taxonomic and phylogenetic reconstruction and has enhanced the sensitivity of AMF identification and quantification (Lekberg et al., 2007; Helgason & Fitter, 2009; Balestrini et al., 2010; Gast et al., 2011). Moreover, significant improvements have been made in the analysis of AMF condition by the high-throughput technology (Margulies et al., 2006). Determining the diversity of AMF became very widespread by using regions of the small ribosomal subunit gene. Due to technology advancements, it can provide the most comprehensive reference sequence data set (Öpik et al., 2010), and the sequencing data can provide detailed analyses on AMF communities among complex habitat types (Öpik et al., 2013). In summary, the application of new technologies will greatly improve the study of AMF communities.

Thus, our study applied the high-throughput sequencing (Illumina platform) to analyze the soil AMF communities in four habitat types, including agricultural arable land, artificial forest land, natural grassland, and bush/wood land, and in contrast to the first two soil habitat types, the last two types were undisturbed (without human interference). All habitat types were located in the Taihang Mountain, which belongs to the semi-arid ecosystem. We aimed to identify the relative importance of soil characteristics on AMF diversity and illustrate the differences in AMF communities among the predominant soil types. The research would be a valuable contribution toward a better understanding on the way human activities have changed the composition of the current AMF communities, and the results would contribute to developeing a more precise guidance on local soil reclamation, vegetation restoration, and the maintenance of biodiversity in semi-arid regions.

## Materials and Methods

### Study area

The research site was located in the south of Taihang Mountain (112°28′–112°30′E, 35°01′–35°03′N), a site which belongs to the semi-arid area of China. The climate in the test area is temperate continental monsoon, with an annual average temperature of 14.3 °C and an average annual sunshine rate of 54%; the elevation gradient of our study sites ranged from 231 to 432 m above sea level. Soil in the study area is cinnamon (main part is similar to ustalf USDA), and the parent rock was composed mainly of sandstone and shale. The habitat types in this study were bush/wood land, forest land, grassland, and arable land. The bush/wood land included mainly *Vitex negundo* L, *Lespedeza bicolor* Turcz and *Ziziphus jujuba* Mill. var. *spinosa* (Bunge) Hu ex H.F. Chow, Forest land included mainly *Quercus variabilis* Bl., *Platycladus orientalis* (L.) Franco, and *Robinia pseudoacacia* L. Dominant herbaceous plants in the grassland were *Setaria viridis* (L.) Beauv., *Artemisia princeps* H. Lév. and Vaniot, *Pennisetum alopecuroides* (L.) Spreng., *Arthraxon hispidus* (Thunb.) Makino, and *Rehmannia glutinosa* (Gaetn.) Libosch. ex Fisch. et Mey. Finally, the prevalent herbaceous plants in the arable land were *Zea mays* L., *Triticum aestivum* L., *Ipomoea batatas* L*.*, *Brassica campestris* L., and *Lycopersicon esculentum* Mill*.*

### Sample collection

In October 2016, soil samples were collected in triplicate at four sites (W1, BW, WL, and F). The sample collection occurred at the root zone of the plant at a soil depth of 5–10 cm (Table 1). Site W1 represented the forest land soil type; site BW had bush/wood soil type; site WL was characterized by grassland soil type; and arable land soil type was represented in site F. These 12 soil samples collected were placed in sterile plastic bags and transported in freezing boxes to the laboratory, and they were stored at −70 °C until further analysis.

**Table 1:** Geochemical characteristics of the soil samples and other information of the site of the present study.

Sample sample	Soil type	Coordinates	pH	Water content (%)	Available nitrogen (mg kg^−1^)	Available phosphorus (mg kg^−1^)	Available potassium (mg kg^−1^)	Clay (%)	Silt (%)	Sand (%)
W1-1	Forest land	35°1′56″N 112°29′1″E	7.32	19.81	155.3	7.8	169.5	47.1	41.5	11.4
W1-2		35°2′16″N 112°28′20″E	7.41	20.24	145.3	6.9	143.4	46.2	44.5	9.3
W1-3		35°2′45″N 112°28′52″E	7.34	18.29	182.3	7.2	162.4	41.2	48.9	9.9
Average			7.36 A	19.44 B	161.0 A	7.3 B	158.4 B	44.9 AB	45.0 A	10.2 C
BW-1	Bush/ wood	35°1′49″N 112°29′14″E	7.53	20.33	141.5	4.7	177.4	37.3	32.1	30.6
BW-2		35°2′3″N 112°29′29″E	7.41	21.14	133.2	4.8	186.4	41.2	31.5	27.3
BW-3		35°2′55″N 112°29′1″E	7.36	23.00	187.3	6.5	160.1	28.3	42.9	28.8
Average			7.43 A	21.49 B	154 A	5.3 B	174.6 B	35.6 BC	35.5 B	28.9 A
WL-1	Grass land	35°1′41″N 112°29′39″E	7.21	16.71	167.4	4.3	129.1	33.2	52.6	14.2
WL-2		35°2′55″N 112°29′12″E	7.34	15.90	144.3	5.1	135.4	26.4	49.6	24
WL-3		35°1′38″N 112°28′58″E	7.42	18.05	132.2	2.4	122.0	37.5	48.9	13.6
Average			7.32 A	16.88 C	148.0 A	3.93 B	128.8 C	32.37 C	50.4 A	17.3 B
F-1	Arable land	35°2′31″N 112°29′55″E	7.27	26.71	177.5	18.7	287.4	55.2	39.5	5.3
F-2		35°2′12″N 112°29′38″E	7.45	23.71	183.5	22.5	254.3	50.1	43.6	6.3
F-3		35°2′43″N 112°29′19″E	7.33	23.95	182.3	30.2	299.1	47.8	47.5	4.7
Average			7.35 A	24.79 A	181.1 A	23.8 A	280.3 A	51.0 A	43.53 AB	5.4 C

**Notes.**

Values (eg. A, B, C) followed by the same letters in the same column was not significantly different (*p* < 0.05).

### Soil geochemical analyses

We analyzed eight different soil factors, including soil pH, water content, available nitrogen (NH${}_{4}^{+}$-N), available potassium (K^+^-K) and phosphate phosphorus (PO${}_{4}^{3-}$-P). Soil pH was examined by a pH meter (PX-KS06; Guangzhou Puxi Instrument, Guangzhou, China). Water content was measured by drying soil method, and the content of soil clay, silt, and sand was performed by using a Malvern Mastersizer (Mastersizer2000; Malvern Instruments, Malvern, UK). The available nitrogen and available potassium were analyzed by an Autoanalyzer (SEAL-AA3; SEAL Analytical, Milwaukee, WI, USA); phosphate phosphorus analyzed by NaHCO_3_ Mo-Sb colorimetric method.

### Molecular analyses DNA extraction

A total of 50 mg soil was used for metagenomic DNA extraction in each sample, using the Fast DNA Isolation Kit (Q-BIOgene; Heidelberg, Germany). The extracts were stored at −20 °C for PCR. 1.0% agarose gels for checking DNA concentration and purity.

### Miseq sequencing step

Using the 18S rRNA gene and primer sets of AMV4.5N Forward 5′-AAGCTCGTAGTT- GAATTTCG-3′ and AMDG R 5′-CCCAACTATCCCTATTAATCAT-3′ to amplify the sequences (from soil DNA extracts), the primer had been reported to be acceptable in several previous studies (Sato et al., 2005). The initial PCR reactions were similar to the existing studies of (Xiao et al., 2016), including :25 µL total volumes, 1–2 µL DNA template, 250 mM dNTPs, 0.25 mM of primer, 1× reaction buffer and 0.5U Phusion DNA Polymerase.

The reactions used a 2720 model Thermal Cycler, and initial PCR amplification was conducted under the steps below: 94 °C for 2-min, then 25 cycles of 30-s denaturation at 94 °C, 30-s annealing at 56 °C, 30-s extension at 72 °C, 5-min extension at 72 °C.The second step PCR used a template, which come from the first 5uL product (without dilution). The second step PCR include: one cycle of 3-min at 94 °C, then 8 cycles of 30-s at 94 °C, 56 °C for 30-s and 72 °C for 30-s, and a 5-min extension at 72 °C. The PCR products were separated by electrophoresis (1.5% agarose gel in 0.5 × TBE) and purified using a gel xxtraction kit (Axygen Biosciences, Corning, NY, USA), then the libraries were sequenced by PE300 sequencing on MiSeq v3 Reagent Kit (Illumina) platform (at Tiny Gene Company, Shanghai).

### Bioinformatics methods

The sequence reads were analyzed by the combination of software Mothur version 1.33.3, UPARSE (USEARCH version v8.1.1756) and R 3.2.2 (R Development Core Team, 2015), the original FASTQ files were demultiplexed through the barcode (Schloss et al., 2009). The PE reads for all samples were merged based on mothur. The low quality contigs were removed based on screen.seqs command by the settings filter (maxambig = 0, minlength = 200, maxlength = 580, the higher threshold can protect some longer sequences, which may be the correct fragment, maxhomop = 8). The decoded data information was aggregated (97% homology) to operational taxonomic units (OTUs) (Edgar, 2013).

BLAST analysis was conducted using the “Nucleotide collection (nr/nt)” database (https://blast.ncbi.nlm.nih.gov/Blast.cgi?PAGE_TYPE=BlastSearch). No threshold was set for *E* values, alignment length and identity settings. For each OTU representative sequence, a list of top BLAST hits was compiled. Uncultured clones were deleted from the list of top hits. The BLAST hit getting the highest score was identifed as the match’s species.

### Statistical analyses

For the alpha-diversity analysis, Mothur version 1.33.3 software (Schloss et al., 2009) was used to analyze the OTU richness, Coverage, Chao, and Shannon’s indices as reported earlier by Schloss et al. (2009). The values of soil properties and diversity parameters were statistically analysed by SPSS V. 19 software (one-way ANOVA) (SPSS; IBM Corp., Armonk, NY, USA).

The clustering method was used with R v. 3.1.1 software to identify the AMF relationship (based on OTU abundance-based). Further, the indicator species analysis was utilized to identify the AMF communities associated with various habitat types (Dufrene & Legendre, 1997).

Using the Canoco software (Canoco for Windows 4.5 package) (Braak & Smilauer, 2002), we utilized Monte Carlo permutation and distance-based redundancy (db-RDA) tests to explain the correlation between soil AMF and geochemical factors. In addition, the heatmap results of the abundance percentages of AMF genera were obtained by Mothur version 1.33.3 software. The raw sequence information has been deposited into the NCBI database (accession number SRP116770).

## Results

### Soil properties

For the eight geochemical factors measured, the arable land obtained the maximum values of water content, available phosphorus and available potassium (site F). Meanwhile, the minimum values of water content and available phosphorus were established in the grassland (site WL). In the bush/wood land (site BW), the maximum values of sand content (average 28.9%), but minimum silt content (35.5%) were established (Table 1).

### AMF diversity data and community composition

In the current study, we have identified a total of 532,841 sequences and 803 OTUs from the total dataset; there were 320,899 sequences belonged to phylum Glomeromycotina (accounting for 60.2%). The number of sequences in each of the samples ranged from 15,095 to 35,206, and the number of AMF OTUs ranged from 52 to 83 (genetic distances of 3%). The OTUs’ coverage in all soil types reached 99% (Table 2). On the basis of the OTU richness calculated by Chao’s index, the grassland observed the greatest AMF value (site WL: 81). Through the analysis of Shannon’s index, we discovered that the largest AMF diversity was also present in the grassland (site WL: 3.49–3.52 with an average value of 3.51), followed by the arable land (site F: 3.38–3.46 with an average value of 3.43), bush/wood land (site BW: 3.38–3.46 with average 3.42), and the forest land soils (site W1: 2.53–3.15 with an average value of 2.87) (Table 2).

**Table 2:** The results of sequence data in the present study.

	Soil type	Number of total sequence	Number of AMF OTUs	Coverage (%)	Chao’s index	Shannon’s index
W1-1	Forest land	26,866	62	99	67	2.93
W1-2		27,298	71	99	73	3.15
W1-3		28,493	60	99	60	2.53
Average					67 A	2.87 B
BW-1	Bush/ wood	31,391	73	99	74	3.46
BW-2		35,206	69	99	70	3.38
BW-3		32,153	67	99	68	3.42
Average					70 A	3.42 A
WL-1	Grass land	29,593	74	99	76	3.52
WL-2		28,148	83	99	87	3.49
WL-3		28,621	76	99	79	3.51
Average					81 A	3.51 A
F-1	Arable land	18,900	52	99	67	3.46
F-2		19,135	62	99	88	3.38
F-3		15,095	54	99	55	3.45
Average					70 A	3.43 A

**Notes.**

The OTUs were defined at the cutoff 3% difference in sequence. Using the one-way analysis of variance (ANOVA) to evaluate statistical significance and results, followed by Tukey’s HSD test. Capital direction symbols (eg. A, B, C) indicate full (5%) significance.

**Figure 1:**
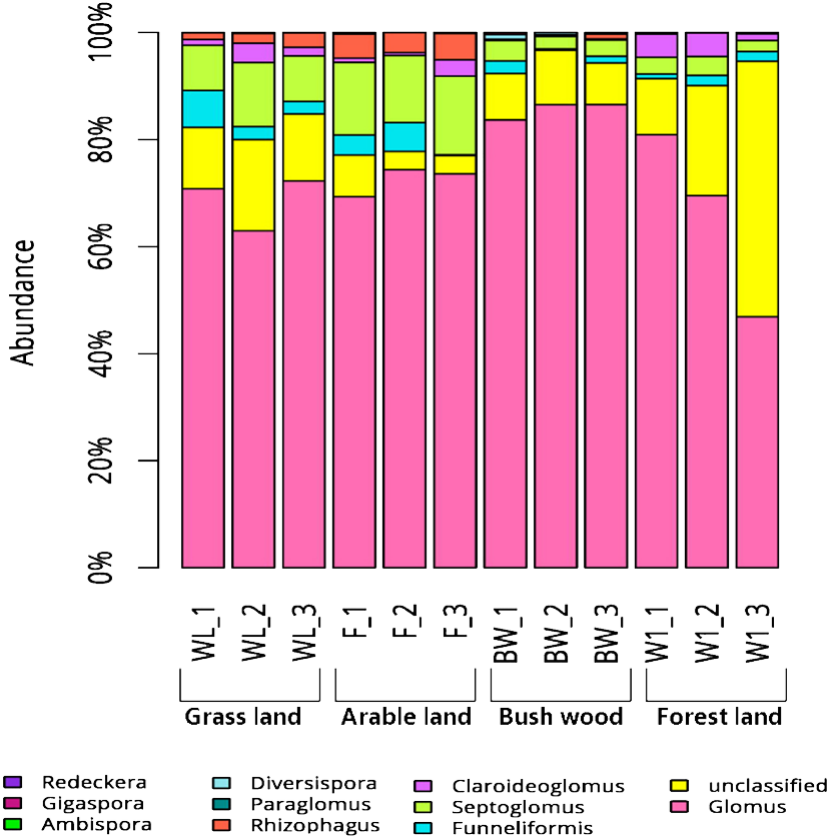
Abundance percentages of AMF genera for all soil samples.

Some variations in AMF community composition at the genus level were also detected among all soil samples. The 119 OTUs that could be classified were affiliated with ten AMF genera, whereas those that could not be identified were assigned as unclassified. The *Glomus* were the most abundant genera in all samples: 60%–75% in grassland, 70%–75% in arable land, 75%–80% in bush/wood, and 50%–70% in forest land. Meanwhile, their levels varied in the different soil types. *Ambispora* were found in all samples, but a greater abundance was detected in the grassland and arable land samples than in those of the bush/wood and forest land soils (Fig. 1).

### Correlation among the three factors (AMF communities, soil types and environmental condition)

**Figure 2:**
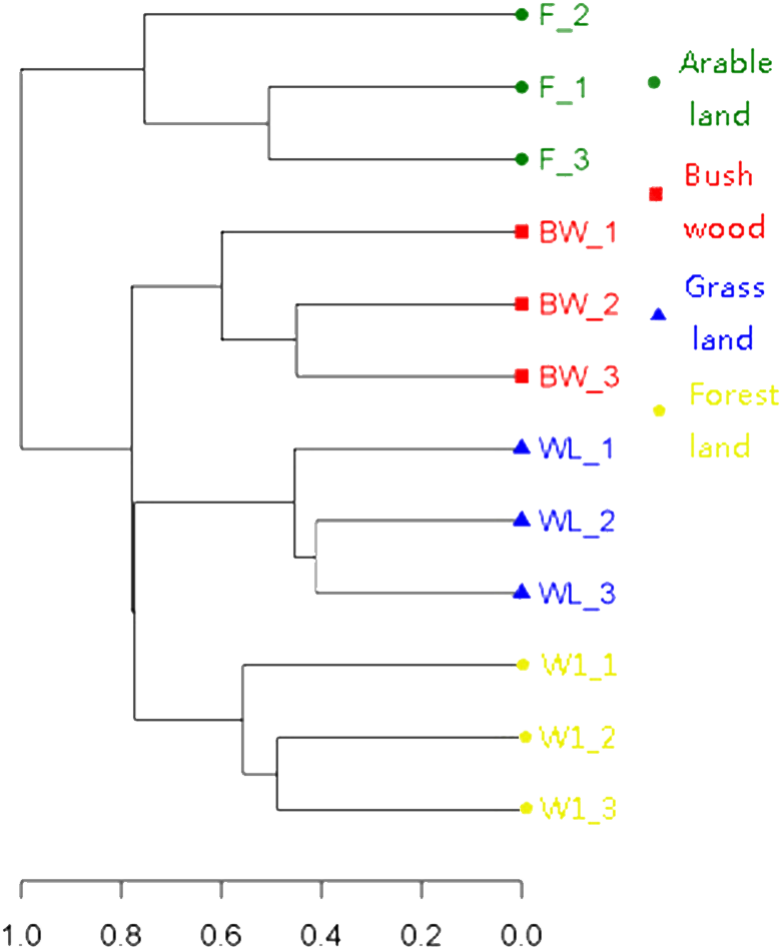
Clustering analysis of AMF communities based on OTU abundance for each soil.

To determine the differences in soil AMF community, the OTU cluster analysis showed that the 12 soil samples were divided into four Soil Types (Fig. 2), and the indicator species analysis revealed that there were 60 AMF indicators (indicator value > 0.25, *p* < 0.05) in this four groups types, it mainly included bush/wood (*Glomus* and *Diversispora* taxa), arable land (*Glomus*, *Septoglomus* and *Rhizophagus* taxa), grassland (*Glomus* and *Septoglomus* taxa), forest land (*Glomus* and *Paraglomus* taxa) (Table S1).

**Figure 3:**
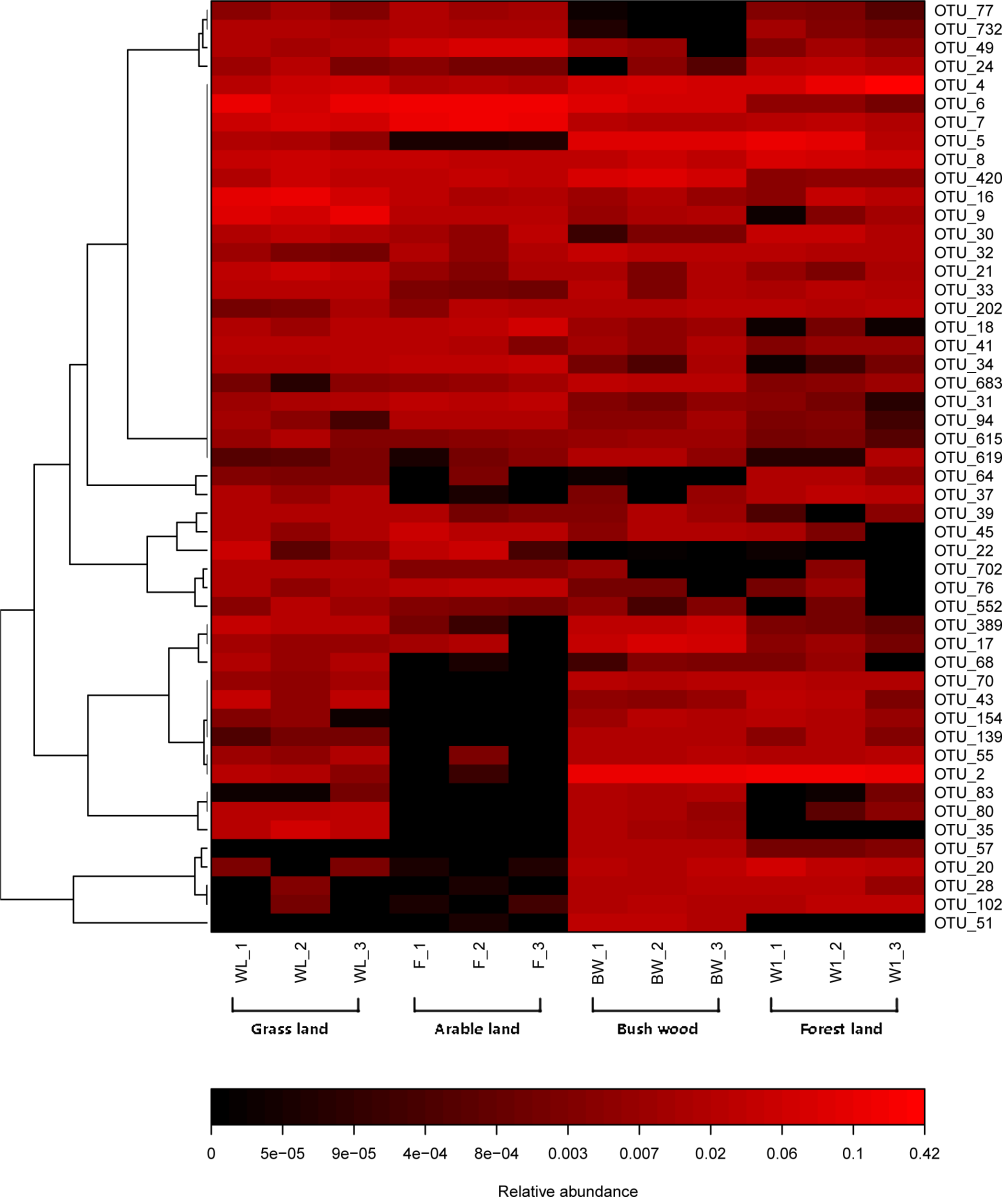
Heat map of top 50 OTUs in all samples. The color intensity (log scale) in each panel shows the percentage of a genus in a sample, referring to color key at the bottom.

The top 50 OTUs of all samples were selected and their abundances were compared by heatmap software. It revealed the relative distributions and abundances of the top 50 OTUs in all samples (Fig. 3). More detailed information about the top 50 OTUs was presented in Table S2. There is also a listing of all AMF OTUs and their closest matches in Table S3.

**Figure 4:**
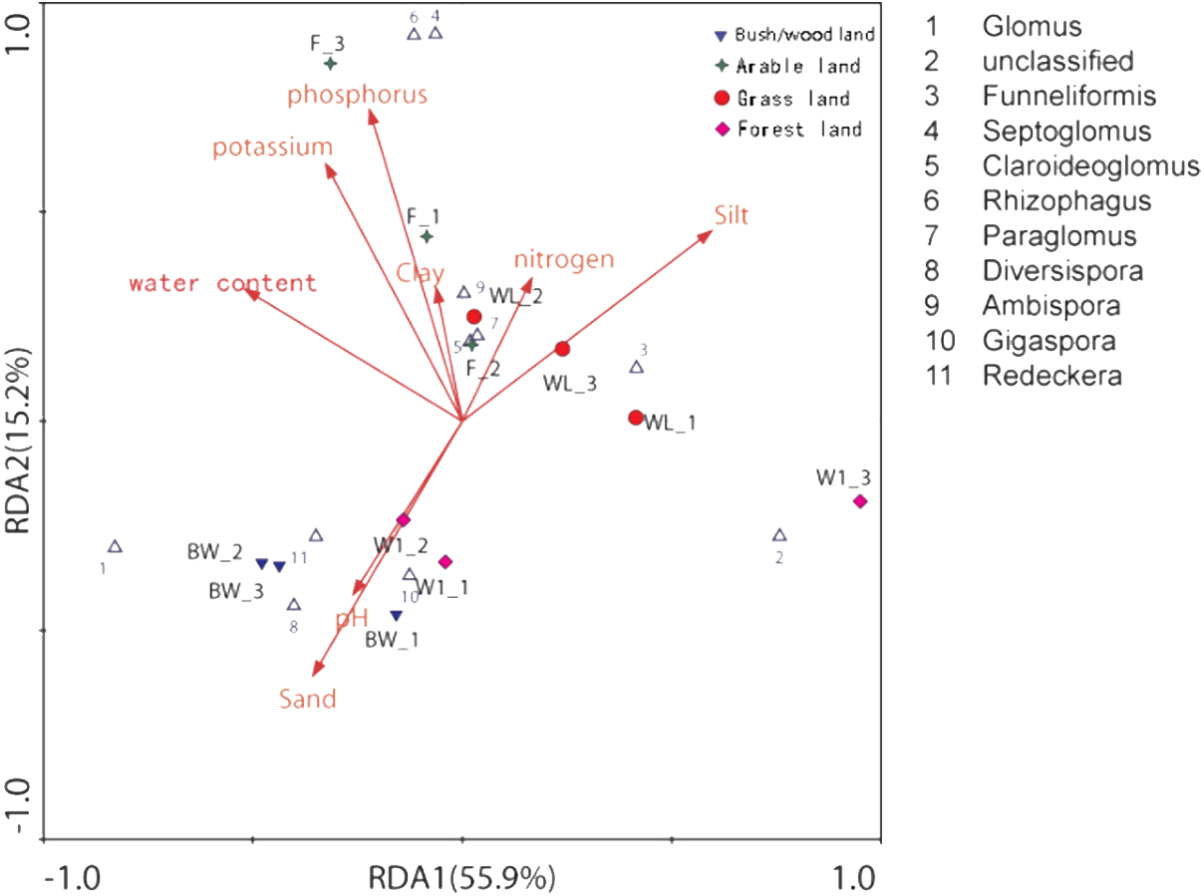
Distance-based redundancy (db-RDA) tests used to interpret the correlations between the AMF communities and environmental properties.

The distance-based redundancy analysis (db-RDA) showed that there was a significant correlation between the combination of eight environmental factors and soil AMF community structure, that 81.9% of the soil community variation was attributed to all environmental factors (Fig. 4 and Table 3). However, using the Monte Carlo permutation test, we found that water content (*r*^2^ = 0.7332, *p* < 0.01), available phosphorus (*r*^2^ = 0.7576, *p* < 0.01), available potassium (*r*^2^ = 0.7973, *p* < 0.01), silt (*r*^2^ = 0.6461, *p* < 0.05), and sand (*r*^2^ = 0.6293, *p* < 0.05) were important properties (Table 3).

**Table 3:** Monte Carlo permutation tests were used to detect the relationship between community composition and soil variables.

	RDA1	RDA2	*r*^2^	*P*-value
pH	−0.2607	−0.4144	0.2053	0.353
Water content	−0.5188	0.3150	0.7332	0.004^**^
Available nitrogen	0.1659	0.3414	0.3096	0.186
Available phosphorus	−0.2218	0.7446	0.7576	0.004^**^
Available potassium	−0.3262	0.6153	0.7973	0.004^**^
Clay	−0.0622	0.3178	0.3611	0.156
Silt	0.5950	0.4549	0.6461	0.015^*^
Sand	−0.3564	−0.6083	0.6293	0.013^*^

**Notes.**

* Correlation is significant at the 0.05 level.

** Correlation is significant at the 0.01 level.

*P*-values based on 999 permutations.

## Discussion

As mentioned earlier, the study area was located in the South Taihang Mountains of China, whose climate characterizes the region as a typical semi-arid climate zone. Under natural conditions, the thin soil layer, low forest coverage and much gravel are the characteristics of this area. Its forest types are mainly dominated by human intervention of *Quercus variabilis* Bl and *Platycladus orientalis* (L.); the vegetation is poor and only limited species could be planted (Zhao, 2007). Thus, improving local soil conditions and promoting plant growth are urgent tasks. However, some information had remained unexplored for the Taihang Mountain area, such as the distribution of AMF communities, the variation of AMF diversity, and the influence of various soil types on AMF composition. Therefore, in this study, we investigated the AMF communities among the predominant soil types in the South Taihang Mountain region. The results could be a valuable reference for improving the local ecological environment.

By analyzing the results of the four different soil types, the research showed that the diversity of AMF communities in undisturbed grassland soil type was greater than that in artificial forest land (Table 2). That was consistent with Öpik et al. (2008), who discovered that rich biological species composition and low external disturbance may lead to higher diversity of rhizosphere AMF of the natural vegetation soil. Our results showed that the value of Shannon’s index in arable land was larger than that in artificial forest land. This outcome might have been caused by the cultivation practices implemented by the farmers, including the application of farmyard manure (food residues, livestock manure, etc.), which increased the number of microbial communities by raising the level of available nutrients (Helgason & Fitter, 2009). Indeed, it is generally accepted that the organic agriculture farming methods are regarded as a useful measure to increase AMF diversity (Aroca, Porcel & Ruiz-Lozano, 2007), and farmers in that region usually apply farmyard manure with cultivation methods that are closed to organic agriculture farming. On the other hand, probably because the growth and reproduction of specific AMF communities requiring particular host plant species, it leads to a less abundant community under a single artificial plantation habitat (Long et al., 2010). In general, human disturbance caused changes in the forest land environment, which reduced the transportation and distribution of AMF communities (Yuan et al., 2008), and the artificial forest land had the lowest AMF diversity in comparison with other land types.

Meanwhile, the results of the sequence data analysis of AMF community composition showed that members of both genera *Septoglomus* and *Glomus* existed in different soil types, including forest land, bush/wood, grassland, and arable land. Nevertheless, the representatives of *Glomus* were identified to be the main genus, and although *Glomus, Diversispora, Septoglomus, Rhizophagus and Paraglomus* were found in soils, only *Glomus* taxa served as indicator species for each habitat. These results are similar to previously published research that confirmed that the species of *Glomus* were the most abundant in the AMF assemblage (Oehl et al., 2005). The influence of certain factors may be the reason why *Glomus* was the dominant members in the AMF assemblage among those of other genera. Some researchers revealed that the species of *Glomus* genus can usually produce large numbers of spores and hypha fragments, which can colonize and extensively spread onto the roots of plants (Öpik et al., 2006). *Glomus* has also a certain resistance in complex environments (Miransari et al., 2008; Bever et al., 2009; Barto et al., 2011). Therefore, these features facilitate the survival and spread of *Glomus* genus members in a semi-arid mountain, and the emergence of this phenomenon is also the result of adaptation to the local ecological environment.

Moreover, our investigation established that water content is a significant factor which has an obvious effect on the AMF communities. Scholars have shown that the variations in the water content can contribute to changes in the physiological status of local AMF and its ecological niche directly (Sieverding, Toro & Mosquera, 1989), probably because the water was essential for the reproductive and metabolic processes. Thus, the water content can indirectly exert an impact on the distribution of AMF communities. In addition, our research also confirmed that there are significant relationships between the available phosphorus, available potassium, and soil AMF community structure. These interactions were most likely attributable to the fact that soil phosphorus may stimulate the spore germination and hyphal growth of AMF (Miranda & Harris, 1994), and the potassium has the ability to increase the infection rate of AMF under drought stress (Wei, 2016). In general, soil nutrients can have on the growth of local AMF communities as the lack of nutrients inhibits the production and separation of spores (Zaller, Frank & Drapela, 2011). Thus, this work confirmed that environmental factors can drive the composition and distribution of AMF communities.

Furthermore, the composition of AMF communities seems to have been strongly influenced by the soil texture distribution, and our results showed that the content of silt and sand were significantly related to the soil AMF community (Table 1). The AMF diversity was higher in the samples from low-clay but high-sand content soil types. The appearance of the result was probably due to the fact that AMF is an aerobic organism, and the lower clay content provided better aeration, which was advantageous for plant root growth and soil humus decomposition, leading also to accelerated fungal propagation (Torrecillas et al., 2014). The research confirmed that AMF communities was negatively correlated with soil clay content.

## Conclusions

In conclusion, this study first delineated the species diversity and composition of AMF communities in Taihang Moutain, China. The members of the *Glomus* genus were predominant in all soil types. The findings also suggested that nutrient composition and soil texture were the most important factors affecting AMF communities. Moreover, there were differences in species diversity and composition of soil AMF communities among different habitat types. These findings shed new light on the characteristics of community structure and drivers of community assembly in AMF in semi-arid mountains, and point to the potential importance of different habitat types on AMF communities.

##  Supplemental Information

10.7717/peerj.4155/supp-1 Table S1 Results of indicator species analysis showing AMF characteristic (indicator value > 0.25, *p*.value < 0.05) in each habitat type

10.7717/peerj.4155/supp-2 Table S2 Detailed information about the dominant 50 OTUs in heat map

10.7717/peerj.4155/supp-3 Table S3 Detailed information about all AMF OTUs

10.7717/peerj.4155/supp-4 Supplemental Information 1 The sequence information of WL-1

10.7717/peerj.4155/supp-5 Supplemental Information 2 The sequence information of WL-2

10.7717/peerj.4155/supp-6 Supplemental Information 3 The sequence information of WL-3

10.7717/peerj.4155/supp-7 Supplemental Information 4 The sequence information of F-1

10.7717/peerj.4155/supp-8 Supplemental Information 5 The sequence information of F-2

10.7717/peerj.4155/supp-9 Supplemental Information 6 The sequence information of F-3

10.7717/peerj.4155/supp-10 Supplemental Information 7 The sequence information of BW-1

10.7717/peerj.4155/supp-11 Supplemental Information 8 The sequence information of BW-2

10.7717/peerj.4155/supp-12 Supplemental Information 9 The sequence information of BW-3

10.7717/peerj.4155/supp-13 Supplemental Information 10 The sequence information of W1-1

10.7717/peerj.4155/supp-14 Supplemental Information 11 The sequence information of W1-2

10.7717/peerj.4155/supp-15 Supplemental Information 12 The sequence information of W1-3

10.7717/peerj.4155/supp-16 Supplemental Information 13 The sequences uploaded to NCBI

## Additional Information and Declarations

### DNA Deposition

The following information was supplied regarding the deposition of DNA sequences: SRA: SRP116770.

### Data Availability

The following information was supplied regarding data availability: The raw sequence information have been deposited into the NCBI database (accession number SRP116770).
