# Supplementary material for: Species diversity and drivers of arbuscular mycorrhizal fungal communities in a semi-arid mountain in China
Source: PeerJ. 2017 Dec 8;5:e4155. doi: 10.7717/peerj.4155 (PMC5724403; doi:10.7717/peerj.4155)
Supplement: Table S1 [file peerj-05-4155-s001.docx]

Table S1 Results of indicator species analysis showing AMF characteristic (indicator value > 0.25, p.value < 0.05) in each habitat type

| OTUID | genus | species | s.bushwood | s.arableland | s.grassland | s.forestland | stat | p.value |
| --- | --- | --- | --- | --- | --- | --- | --- | --- |
| OTU_7 | Glomus | Glomus_sp._C_2-25 | 0 | 1 | 0 | 0 | 0.804 | 0.0095 |
| OTU_45 | Glomus | Glomus_indicum | 0 | 1 | 0 | 0 | 0.764 | 0.0143 |
| OTU_94 | Glomus | Glomus_indicum | 0 | 1 | 0 | 0 | 0.728 | 0.0169 |
| OTU_683 | Glomus | Glomus_sp._MO-G6 | 1 | 0 | 0 | 0 | 0.804 | 0.0176 |
| OTU_17 | Glomus | Glomus_sp._VeGlo18 | 1 | 0 | 0 | 0 | 0.878 | 0.0177 |
| OTU_51 | Glomus | Glomus_sp._Glo38 | 1 | 0 | 0 | 0 | 1.000 | 0.0177 |
| OTU_57 | Glomus | Glomus_sp._Glo50 | 1 | 0 | 0 | 0 | 0.928 | 0.0177 |
| OTU_60 | Glomus | Glomus_sp._Glo40 | 1 | 0 | 0 | 0 | 0.998 | 0.0177 |
| OTU_61 | Glomus | Glomus_sp._MO-G8 | 1 | 0 | 0 | 0 | 0.883 | 0.0177 |
| OTU_83 | Glomus | Glomus_sp._VeGloB | 1 | 0 | 0 | 0 | 0.968 | 0.0177 |
| OTU_343 | Glomus | Glomus_sp._Glo50 | 1 | 0 | 0 | 0 | 1.000 | 0.0177 |
| OTU_420 | Glomus | Glomus_sp._MO-G8 | 1 | 0 | 0 | 0 | 0.691 | 0.0177 |
| OTU_586 | Glomus | Glomus_sp._MO-G6 | 1 | 0 | 0 | 0 | 0.986 | 0.0177 |
| OTU_6 | Glomus | Glomus_sp._M01 | 0 | 1 | 0 | 0 | 0.758 | 0.0179 |
| OTU_18 | Glomus | Glomus_sp._Glo45 | 0 | 1 | 0 | 0 | 0.808 | 0.0179 |
| OTU_31 | Septoglomus | Septoglomus_viscosum | 0 | 1 | 0 | 0 | 0.830 | 0.0179 |
| OTU_34 | Rhizophagus | Rhizophagus_intraradices | 0 | 1 | 0 | 0 | 0.821 | 0.0179 |
| OTU_49 | Septoglomus | Septoglomus_constrictum | 0 | 1 | 0 | 0 | 0.860 | 0.0179 |
| OTU_76 | Glomus | Glomus_sp._C_2-2 | 0 | 1 | 0 | 0 | 0.853 | 0.0179 |
| OTU_86 | Septoglomus | Septoglomus_viscosum | 0 | 1 | 0 | 0 | 0.948 | 0.0179 |
| OTU_237 | Glomus | Glomus_sp._NBR_PP1 | 0 | 1 | 0 | 0 | 1.000 | 0.0179 |
| OTU_246 | Glomus | Glomus_sp._MS | 0 | 1 | 0 | 0 | 0.916 | 0.0179 |
| OTU_772 |  | Glomeromycota_sp._WR856-B | 0 | 1 | 0 | 0 | 0.999 | 0.0179 |
| OTU_9 | Glomus | Glomus_sp._Glo38 | 0 | 0 | 1 | 0 | 0.843 | 0.0183 |
| OTU_16 |  | Glomeromycota_sp._AB-2014 | 0 | 0 | 1 | 0 | 0.791 | 0.0183 |
| OTU_21 | Septoglomus | Septoglomus_viscosum | 0 | 0 | 1 | 0 | 0.817 | 0.0183 |
| OTU_35 | Glomus | Glomus_sp._MSLA-8 | 0 | 0 | 1 | 0 | 0.888 | 0.0183 |
| OTU_68 | Glomus | Glomus_sp._C_2-2 | 0 | 0 | 1 | 0 | 0.817 | 0.0183 |
| OTU_80 | Glomus | Glomus_sp._Glo2 | 0 | 0 | 1 | 0 | 0.823 | 0.0183 |
| OTU_84 | Glomus | Glomus_sp._Glo14 | 0 | 0 | 1 | 0 | 0.807 | 0.0183 |
| OTU_91 | Glomus | Glomus_sp._5014b25.Llao5 | 0 | 0 | 1 | 0 | 0.983 | 0.0183 |
| OTU_100 | Glomus | Glomus_sp._Glo40 | 0 | 0 | 1 | 0 | 0.953 | 0.0183 |
| OTU_220 | Glomus | Glomus_sp._NBR_PP1 | 0 | 0 | 1 | 0 | 1.000 | 0.0183 |
| OTU_263 | Glomus | Glomus_sp._Glo40 | 0 | 0 | 1 | 0 | 0.924 | 0.0183 |
| OTU_583 | Glomus | Glomus_sp._Glo16 | 0 | 0 | 1 | 0 | 1.000 | 0.0183 |
| OTU_702 | Glomus | Glomus_sp._Glo38 | 0 | 0 | 1 | 0 | 0.814 | 0.0183 |
| OTU_2 | Glomus | Glomus_sp._0904-1 | 0 | 0 | 0 | 1 | 0.763 | 0.0193 |
| OTU_64 |  | Glomeromycota_sp._MIB_8859 | 0 | 0 | 0 | 1 | 0.889 | 0.0193 |
| OTU_166 | Glomus | Glomus_sp._C_3-10 | 0 | 0 | 0 | 1 | 0.951 | 0.0193 |
| OTU_238 | Paraglomus | Paraglomus_sp._BSTA-3 | 0 | 0 | 0 | 1 | 0.971 | 0.0193 |
| OTU_77 | Septoglomus | Septoglomus_viscosum | 0 | 1 | 0 | 0 | 0.772 | 0.0280 |
| OTU_101 | Glomus | Glomus_sp._PSAMG4 | 0 | 0 | 0 | 1 | 0.870 | 0.0341 |
| OTU_92 | Diversispora | Diversispora_sp._S-MA-3 | 1 | 0 | 0 | 0 | 0.907 | 0.0343 |
| OTU_145 | Glomus | Glomus_sp._Glo38 | 1 | 0 | 0 | 0 | 0.892 | 0.0347 |
| OTU_69 |  | Glomeromycota_sp._AB-2014 | 0 | 0 | 1 | 0 | 0.899 | 0.0348 |
| OTU_8 | Glomus | Glomus_sp._MO-G6 | 0 | 0 | 0 | 1 | 0.586 | 0.0351 |
| OTU_24 | Glomus | Glomus_sp._BSLA-7 | 0 | 0 | 0 | 1 | 0.791 | 0.0351 |
| OTU_552 | Septoglomus | Septoglomus_viscosum | 0 | 0 | 1 | 0 | 0.835 | 0.0364 |
| OTU_4 |  | Glomeromycota_sp._AB-2014 | 0 | 0 | 0 | 1 | 0.781 | 0.0367 |
| OTU_102 |  | Glomeromycota_sp._MIB_8856 | 0 | 0 | 0 | 1 | 0.799 | 0.0367 |
| OTU_169 | Glomus | Glomus_sp._C_3-10 | 0 | 0 | 1 | 0 | 0.806 | 0.0368 |
| OTU_619 | Glomus | Glomus_sp._VeGlo10 | 1 | 0 | 0 | 0 | 0.778 | 0.0368 |
| OTU_41 | Glomus | Glomus_sp._Glo16 | 0 | 0 | 1 | 0 | 0.698 | 0.0369 |
| OTU_37 | Glomus | Glomus_sp._Glo3b | 0 | 0 | 0 | 1 | 0.800 | 0.0371 |
| OTU_32 | Glomus | Glomus_perpusillum | 1 | 0 | 0 | 0 | 0.707 | 0.0373 |
| OTU_679 | Glomus | Glomus_sp._Glo25 | 0 | 0 | 0 | 1 | 0.802 | 0.0378 |
| OTU_723 | Glomus | Glomus_sp._0904-1 | 0 | 0 | 0 | 1 | 0.771 | 0.0388 |

Note: 1 represent the indicator species of this group.
