# Supplementary material for: Species diversity and drivers of arbuscular mycorrhizal fungal communities in a semi-arid mountain in China
Source: PeerJ. 2017 Dec 8;5:e4155. doi: 10.7717/peerj.4155 (PMC5724403; doi:10.7717/peerj.4155)
Supplement: Table S3 [file peerj-05-4155-s003.doc]

**Table S3 Detailed information about all AMF OTUs .**

| OTU name | WL_1 | WL_2 | WL_3 | F_1 | F_2 | F_3 | BW_1 | BW_2 | BW_3 | W1_1 | W1_2 | W1_3 | OTU  size | Taxonomy |
| --- | --- | --- | --- | --- | --- | --- | --- | --- | --- | --- | --- | --- | --- | --- |
| OTU_2 | 623 | 281 | 126 | 0 | 3 | 0 | 3622 | 4855 | 4279 | 6647 | 5348 | 4102 | 29886 | sk__Eukaryota;Opisthokonta;k__Fungi;p__Glomeromycota;c__Glomeromycetes;o__Glomerales;f__Glomeraceae;g__Glomus;s__Glomus_sp._0904-1; |
| OTU_9 | 2416 | 1659 | 3269 | 378 | 483 | 338 | 203 | 312 | 308 | 1 | 86 | 228 | 9681 | sk__Eukaryota;Opisthokonta;k__Fungi;p__Glomeromycota;c__Glomeromycetes;o__Glomerales;f__Glomeraceae;g__Glomus;s__Glomus_sp._Glo38; |
| OTU_16 | 2462 | 3113 | 1729 | 549 | 179 | 187 | 214 | 402 | 195 | 116 | 1215 | 804 | 11165 | sk__Eukaryota;Opisthokonta;k__Fungi;p__Glomeromycota;unclassified_Glomeromycota;s__Glomeromycota_sp._AB-2014; |
| OTU_22 | 1448 | 18 | 135 | 662 | 1005 | 5 | 0 | 1 | 0 | 1 | 0 | 0 | 3275 | sk__Eukaryota;Opisthokonta;k__Fungi;p__Glomeromycota;c__Glomeromycetes;o__Glomerales;f__Glomeraceae;g__Funneliformis;s__Funneliformis_mosseae; |
| OTU_389 | 1193 | 749 | 773 | 19 | 3 | 0 | 1092 | 1315 | 1561 | 67 | 36 | 20 | 6828 | sk__Eukaryota;Opisthokonta;k__Fungi;p__Glomeromycota;c__Glomeromycetes;o__Glomerales;f__Glomeraceae;g__Glomus;s__Glomus_sp._Glo7; |
| OTU_6 | 3742 | 1734 | 3254 | 4168 | 4655 | 3688 | 2644 | 2091 | 2143 | 139 | 151 | 29 | 28438 | sk__Eukaryota;Opisthokonta;k__Fungi;p__Glomeromycota;c__Glomeromycetes;o__Glomerales;f__Glomeraceae;g__Glomus;s__Glomus_sp._M01; |
| OTU_18 | 456 | 197 | 577 | 503 | 596 | 941 | 228 | 186 | 245 | 1 | 30 | 1 | 3961 | sk__Eukaryota;Opisthokonta;k__Fungi;p__Glomeromycota;c__Glomeromycetes;o__Glomerales;f__Glomeraceae;g__Glomus;s__Glomus_sp._Glo45; |
| OTU_43 | 1163 | 153 | 957 | 0 | 0 | 0 | 164 | 140 | 194 | 1008 | 765 | 74 | 4618 | sk__Eukaryota;Opisthokonta;k__Fungi;p__Glomeromycota;c__Glomeromycetes;o__Glomerales;f__Glomeraceae;g__Glomus;s__Glomus_sp._MS_Appelhans_246; |
| OTU_32 | 218 | 70 | 37 | 205 | 110 | 181 | 1196 | 761 | 892 | 678 | 348 | 283 | 4979 | sk__Eukaryota;Opisthokonta;k__Fungi;p__Glomeromycota;c__Glomeromycetes;o__Glomerales;f__Glomeraceae;g__Glomus;s__Glomus_perpusillum; |
| OTU_615 | 171 | 392 | 80 | 61 | 76 | 79 | 201 | 241 | 245 | 45 | 58 | 15 | 1664 | sk__Eukaryota;Opisthokonta;k__Fungi;p__Glomeromycota;c__Glomeromycetes;o__Glomerales;f__Glomeraceae;g__Glomus;s__Glomus_sp._MO-G8; |
| OTU_420 | 469 | 1417 | 794 | 657 | 907 | 483 | 2078 | 2657 | 2013 | 129 | 157 | 143 | 11904 | sk__Eukaryota;Opisthokonta;k__Fungi;p__Glomeromycota;c__Glomeromycetes;o__Glomerales;f__Glomeraceae;g__Glomus;s__Glomus_sp._MO-G8; |
| OTU_91 | 234 | 262 | 283 | 0 | 0 | 0 | 0 | 0 | 31 | 0 | 0 | 0 | 810 | sk__Eukaryota;Opisthokonta;k__Fungi;p__Glomeromycota;c__Glomeromycetes;o__Glomerales;f__Glomeraceae;g__Glomus;s__Glomus_sp._5014b25.Llao5; |
| OTU_80 | 617 | 568 | 823 | 0 | 0 | 0 | 422 | 314 | 188 | 0 | 18 | 123 | 3073 | sk__Eukaryota;Opisthokonta;k__Fungi;p__Glomeromycota;c__Glomeromycetes;o__Glomerales;f__Glomeraceae;g__Glomus;s__Glomus_sp._Glo2; |
| OTU_70 | 173 | 154 | 232 | 0 | 0 | 0 | 753 | 527 | 819 | 589 | 435 | 443 | 4125 | sk__Eukaryota;Opisthokonta;k__Fungi;p__Glomeromycota;c__Glomeromycetes;o__Glomerales;f__Glomeraceae;g__Glomus;s__Glomus_sp._0904-1; |
| OTU_33 | 593 | 659 | 538 | 44 | 25 | 14 | 737 | 84 | 404 | 234 | 526 | 518 | 4376 | sk__Eukaryota;Opisthokonta;k__Fungi;p__Glomeromycota;c__Glomeromycetes;o__Glomerales;f__Glomeraceae;g__Funneliformis;s__Funneliformis_sp._MAY-2012b; |
| OTU_4 | 648 | 1323 | 1739 | 309 | 396 | 161 | 1914 | 2434 | 1892 | 1733 | 2863 | 11550 | 26962 | sk__Eukaryota;Opisthokonta;k__Fungi;p__Glomeromycota;unclassified_Glomeromycota;s__Glomeromycota_sp._AB-2014; |
| OTU_5 | 431 | 254 | 132 | 1 | 1 | 1 | 2395 | 2708 | 2563 | 3247 | 2399 | 774 | 14906 | sk__Eukaryota;Opisthokonta;k__Fungi;p__Glomeromycota;c__Glomeromycetes;o__Glomerales;f__Glomeraceae;g__Glomus;s__Glomus_sp._Glo9; |
| OTU_49 | 494 | 210 | 296 | 899 | 1332 | 1035 | 255 | 200 | 0 | 96 | 216 | 154 | 5187 | sk__Eukaryota;Opisthokonta;k__Fungi;p__Glomeromycota;c__Glomeromycetes;o__Glomerales;f__Glomeraceae;g__Septoglomus;s__Septoglomus_constrictum; |
| OTU_732 | 456 | 296 | 250 | 273 | 169 | 142 | 2 | 0 | 0 | 233 | 89 | 40 | 1950 | sk__Eukaryota;Opisthokonta;k__Fungi;p__Glomeromycota;c__Glomeromycetes;o__Glomerales;f__Glomeraceae;g__Septoglomus;s__Septoglomus_viscosum; |
| OTU_7 | 1485 | 2046 | 1588 | 3363 | 3979 | 2870 | 790 | 535 | 539 | 742 | 905 | 516 | 19358 | sk__Eukaryota;Opisthokonta;k__Fungi;p__Glomeromycota;c__Glomeromycetes;o__Glomerales;f__Glomeraceae;g__Glomus;s__Glomus_sp._C_2-25; |
| OTU_77 | 121 | 213 | 89 | 313 | 139 | 116 | 1 | 0 | 0 | 97 | 66 | 16 | 1171 | sk__Eukaryota;Opisthokonta;k__Fungi;p__Glomeromycota;c__Glomeromycetes;o__Glomerales;f__Glomeraceae;g__Septoglomus;s__Septoglomus_viscosum; |
| OTU_31 | 212 | 258 | 304 | 640 | 404 | 574 | 97 | 39 | 166 | 107 | 43 | 2 | 2846 | sk__Eukaryota;Opisthokonta;k__Fungi;p__Glomeromycota;c__Glomeromycetes;o__Glomerales;f__Glomeraceae;g__Septoglomus;s__Septoglomus_viscosum; |
| OTU_76 | 306 | 134 | 239 | 411 | 669 | 568 | 52 | 49 | 0 | 34 | 206 | 0 | 2668 | sk__Eukaryota;Opisthokonta;k__Fungi;p__Glomeromycota;c__Glomeromycetes;o__Glomerales;f__Glomeraceae;g__Glomus;s__Glomus_sp._C_2-2; |
| OTU_21 | 929 | 1482 | 1010 | 120 | 73 | 136 | 270 | 93 | 352 | 165 | 67 | 272 | 4969 | sk__Eukaryota;Opisthokonta;k__Fungi;p__Glomeromycota;c__Glomeromycetes;o__Glomerales;f__Glomeraceae;g__Septoglomus;s__Septoglomus_viscosum; |
| OTU_30 | 326 | 1019 | 461 | 148 | 97 | 463 | 3 | 84 | 68 | 1167 | 1201 | 363 | 5400 | sk__Eukaryota;Opisthokonta;k__Fungi;p__Glomeromycota;c__Glomeromycetes;o__Glomerales;f__Claroideoglomeraceae;g__Claroideoglomus;s__Claroideoglomus_sp._2_GS-2015; |
| OTU_100 | 150 | 58 | 141 | 0 | 0 | 0 | 0 | 0 | 0 | 0 | 33 | 0 | 382 | sk__Eukaryota;Opisthokonta;k__Fungi;p__Glomeromycota;c__Glomeromycetes;o__Glomerales;f__Glomeraceae;g__Glomus;s__Glomus_sp._Glo40; |
| OTU_674 | 17 | 8 | 3 | 1 | 18 | 12 | 0 | 0 | 0 | 11 | 11 | 0 | 81 | sk__Eukaryota;Opisthokonta;k__Fungi;p__Glomeromycota;c__Glomeromycetes;o__Glomerales;f__Glomeraceae;g__Septoglomus;s__Septoglomus_viscosum; |
| OTU_702 | 278 | 440 | 463 | 65 | 74 | 46 | 191 | 0 | 0 | 0 | 123 | 0 | 1680 | sk__Eukaryota;Opisthokonta;k__Fungi;p__Glomeromycota;c__Glomeromycetes;o__Glomerales;f__Glomeraceae;g__Glomus;s__Glomus_sp._Glo38; |
| OTU_35 | 556 | 1652 | 859 | 0 | 0 | 0 | 434 | 267 | 235 | 0 | 0 | 0 | 4003 | sk__Eukaryota;Opisthokonta;k__Fungi;p__Glomeromycota;c__Glomeromycetes;o__Glomerales;f__Glomeraceae;g__Glomus;s__Glomus_sp._MSLA-8; |
| OTU_34 | 369 | 336 | 523 | 646 | 677 | 705 | 48 | 16 | 298 | 1 | 7 | 52 | 3678 | sk__Eukaryota;Opisthokonta;k__Fungi;p__Glomeromycota;c__Glomeromycetes;o__Glomerales;f__Glomeraceae;g__Rhizophagus;s__Rhizophagus_intraradices; |
| OTU_39 | 457 | 408 | 461 | 316 | 26 | 49 | 90 | 431 | 235 | 12 | 0 | 117 | 2602 | sk__Eukaryota;Opisthokonta;k__Fungi;p__Glomeromycota;c__Glomeromycetes;o__Glomerales;f__Glomeraceae;g__Glomus;s__Glomus_sp._Glo14; |
| OTU_41 | 700 | 526 | 757 | 369 | 211 | 46 | 237 | 180 | 322 | 85 | 174 | 187 | 3794 | sk__Eukaryota;Opisthokonta;k__Fungi;p__Glomeromycota;c__Glomeromycetes;o__Glomerales;f__Glomeraceae;g__Glomus;s__Glomus_sp._Glo16; |
| OTU_69 | 184 | 255 | 40 | 0 | 0 | 0 | 22 | 21 | 0 | 0 | 73 | 0 | 595 | sk__Eukaryota;Opisthokonta;k__Fungi;p__Glomeromycota;unclassified_Glomeromycota;s__Glomeromycota_sp._AB-2014; |
| OTU_81 | 100 | 48 | 120 | 0 | 3 | 0 | 77 | 2 | 104 | 4 | 1 | 101 | 560 | sk__Eukaryota;Opisthokonta;k__Fungi;p__Glomeromycota;c__Glomeromycetes;o__Glomerales;f__Glomeraceae;g__Glomus;s__Glomus_sp._MO-G6; |
| OTU_583 | 239 | 152 | 290 | 0 | 0 | 0 | 0 | 0 | 0 | 0 | 0 | 0 | 681 | sk__Eukaryota;Opisthokonta;k__Fungi;p__Glomeromycota;c__Glomeromycetes;o__Glomerales;f__Glomeraceae;g__Glomus;s__Glomus_sp._Glo16; |
| OTU_114 | 248 | 23 | 0 | 0 | 15 | 46 | 23 | 61 | 43 | 0 | 47 | 48 | 554 | sk__Eukaryota;Opisthokonta;k__Fungi;p__Glomeromycota;c__Glomeromycetes;o__Glomerales;f__Glomeraceae;g__Glomus;s__Glomus_sp._Glo56; |
| OTU_8 | 1204 | 1503 | 1079 | 773 | 630 | 499 | 1167 | 1811 | 947 | 1912 | 1750 | 1469 | 14744 | sk__Eukaryota;Opisthokonta;k__Fungi;p__Glomeromycota;c__Glomeromycetes;o__Glomerales;f__Glomeraceae;g__Glomus;s__Glomus_sp._MO-G6; |
| OTU_17 | 231 | 165 | 163 | 157 | 193 | 0 | 1256 | 2347 | 2094 | 125 | 209 | 34 | 6974 | sk__Eukaryota;Opisthokonta;k__Fungi;p__Glomeromycota;c__Glomeromycetes;o__Glomerales;f__Glomeraceae;g__Glomus;s__Glomus_sp._VeGlo18; |
| OTU_37 | 466 | 159 | 280 | 0 | 1 | 0 | 73 | 0 | 201 | 315 | 1009 | 629 | 3133 | sk__Eukaryota;Opisthokonta;k__Fungi;p__Glomeromycota;c__Glomeromycetes;o__Glomerales;f__Glomeraceae;g__Glomus;s__Glomus_sp._Glo3b; |
| OTU_55 | 207 | 141 | 404 | 0 | 43 | 0 | 457 | 649 | 653 | 265 | 403 | 708 | 3930 | sk__Eukaryota;Opisthokonta;k__Fungi;p__Glomeromycota;c__Glomeromycetes;o__Glomerales;f__Glomeraceae;g__Glomus;s__Glomus_sp._Glo3b; |
| OTU_68 | 370 | 163 | 261 | 0 | 1 | 0 | 6 | 100 | 66 | 73 | 158 | 0 | 1198 | sk__Eukaryota;Opisthokonta;k__Fungi;p__Glomeromycota;c__Glomeromycetes;o__Glomerales;f__Glomeraceae;g__Glomus;s__Glomus_sp._C_2-2; |
| OTU_45 | 397 | 136 | 397 | 943 | 437 | 405 | 138 | 461 | 314 | 254 | 58 | 0 | 3940 | sk__Eukaryota;Opisthokonta;k__Fungi;p__Glomeromycota;c__Glomeromycetes;o__Glomerales;f__Glomeraceae;g__Glomus;s__Glomus_indicum; |
| OTU_84 | 166 | 273 | 193 | 0 | 0 | 22 | 126 | 106 | 37 | 0 | 0 | 61 | 984 | sk__Eukaryota;Opisthokonta;k__Fungi;p__Glomeromycota;c__Glomeromycetes;o__Glomerales;f__Glomeraceae;g__Glomus;s__Glomus_sp._Glo14; |
| OTU_61 | 80 | 0 | 1 | 0 | 1 | 0 | 134 | 287 | 139 | 0 | 0 | 55 | 697 | sk__Eukaryota;Opisthokonta;k__Fungi;p__Glomeromycota;c__Glomeromycetes;o__Glomerales;f__Glomeraceae;g__Glomus;s__Glomus_sp._MO-G8; |
| OTU_96 | 59 | 144 | 191 | 0 | 0 | 0 | 0 | 181 | 0 | 0 | 0 | 0 | 575 | sk__Eukaryota;Opisthokonta;k__Fungi;p__Glomeromycota;c__Glomeromycetes;o__Glomerales;f__Glomeraceae;g__Glomus;s__Glomus_sp._Glo16; |
| OTU_263 | 192 | 102 | 62 | 0 | 0 | 0 | 0 | 1 | 0 | 0 | 43 | 14 | 414 | sk__Eukaryota;Opisthokonta;k__Fungi;p__Glomeromycota;c__Glomeromycetes;o__Glomerales;f__Glomeraceae;g__Glomus;s__Glomus_sp._Glo40; |
| OTU_94 | 239 | 108 | 9 | 197 | 286 | 198 | 119 | 147 | 270 | 57 | 86 | 7 | 1723 | sk__Eukaryota;Opisthokonta;k__Fungi;p__Glomeromycota;c__Glomeromycetes;o__Glomerales;f__Glomeraceae;g__Glomus;s__Glomus_indicum; |
| OTU_54 | 245 | 55 | 89 | 0 | 0 | 0 | 0 | 116 | 40 | 294 | 114 | 0 | 953 | sk__Eukaryota;Opisthokonta;k__Fungi;p__Glomeromycota;c__Glomeromycetes;o__Glomerales;f__Glomeraceae;g__Glomus;s__Glomus_sp._MS; |
| OTU_664 | 70 | 33 | 3 | 6 | 9 | 2 | 0 | 0 | 0 | 5 | 2 | 0 | 130 | sk__Eukaryota;Opisthokonta;k__Fungi;p__Glomeromycota;c__Glomeromycetes;o__Glomerales;f__Glomeraceae;g__Septoglomus;s__Septoglomus_viscosum; |
| OTU_552 | 133 | 748 | 195 | 57 | 48 | 20 | 163 | 10 | 93 | 0 | 34 | 0 | 1501 | sk__Eukaryota;Opisthokonta;k__Fungi;p__Glomeromycota;c__Glomeromycetes;o__Glomerales;f__Glomeraceae;g__Septoglomus;s__Septoglomus_viscosum; |
| OTU_24 | 194 | 658 | 53 | 76 | 37 | 22 | 0 | 149 | 16 | 765 | 945 | 299 | 3214 | sk__Eukaryota;Opisthokonta;k__Fungi;p__Glomeromycota;c__Glomeromycetes;o__Glomerales;f__Glomeraceae;g__Glomus;s__Glomus_sp._BSLA-7; |
| OTU_169 | 112 | 73 | 188 | 0 | 2 | 29 | 0 | 0 | 0 | 41 | 0 | 98 | 543 | sk__Eukaryota;Opisthokonta;k__Fungi;p__Glomeromycota;c__Glomeromycetes;o__Glomerales;f__Glomeraceae;g__Glomus;s__Glomus_sp._C_3-10; |
| OTU_683 | 46 | 2 | 104 | 90 | 122 | 118 | 973 | 686 | 639 | 80 | 122 | 195 | 3177 | sk__Eukaryota;Opisthokonta;k__Fungi;p__Glomeromycota;c__Glomeromycetes;o__Glomerales;f__Glomeraceae;g__Glomus;s__Glomus_sp._MO-G6; |
| OTU_368 | 50 | 10 | 96 | 11 | 6 | 12 | 0 | 25 | 0 | 0 | 0 | 0 | 210 | sk__Eukaryota;Opisthokonta;k__Fungi;p__Glomeromycota;c__Glomeromycetes;o__Glomerales;f__Glomeraceae;g__Septoglomus;s__Septoglomus_viscosum; |
| OTU_480 | 17 | 0 | 0 | 0 | 0 | 0 | 0 | 0 | 0 | 0 | 0 | 0 | 17 | sk__Eukaryota;Opisthokonta;k__Fungi;p__Glomeromycota;c__Glomeromycetes;o__Glomerales;f__Glomeraceae;g__Glomus;s__Glomus_sp._MS06-1; |
| OTU_154 | 95 | 155 | 1 | 0 | 0 | 0 | 222 | 694 | 502 | 607 | 415 | 188 | 2879 | sk__Eukaryota;Opisthokonta;k__Fungi;p__Glomeromycota;c__Glomeromycetes;o__Glomerales;f__Glomeraceae;g__Glomus;s__Glomus_sp._Glo9; |
| OTU_20 | 76 | 0 | 76 | 1 | 0 | 1 | 805 | 523 | 967 | 1725 | 838 | 593 | 5605 | sk__Eukaryota;Opisthokonta;k__Fungi;p__Glomeromycota;c__Glomeromycetes;o__Glomerales;f__Glomeraceae;g__Glomus;s__Glomus_sp._0904-1; |
| OTU_202 | 44 | 75 | 257 | 83 | 379 | 221 | 401 | 425 | 626 | 624 | 317 | 573 | 4025 | sk__Eukaryota;Opisthokonta;k__Fungi;p__Glomeromycota;c__Glomeromycetes;o__Glomerales;f__Glomeraceae;g__Glomus;s__Glomus_perpusillum; |
| OTU_64 | 86 | 70 | 65 | 0 | 45 | 0 | 1 | 0 | 0 | 388 | 489 | 154 | 1298 | sk__Eukaryota;Opisthokonta;k__Fungi;p__Glomeromycota;unclassified_Glomeromycota;s__Glomeromycota_sp._MIB_8859; |
| OTU_755 | 17 | 2 | 13 | 0 | 0 | 0 | 6 | 3 | 8 | 19 | 23 | 3 | 94 | sk__Eukaryota;Opisthokonta;k__Fungi;p__Glomeromycota;unclassified_Glomeromycota;s__Glomeromycota_sp._8530; |
| OTU_320 | 14 | 33 | 0 | 0 | 0 | 14 | 0 | 0 | 0 | 0 | 0 | 0 | 61 | sk__Eukaryota;Opisthokonta;k__Fungi;p__Glomeromycota;c__Glomeromycetes;o__Paraglomerales;f__Paraglomeraceae;g__Paraglomus;s__Paraglomus_sp._MSLA-9; |
| OTU_619 | 15 | 16 | 69 | 1 | 36 | 68 | 357 | 493 | 180 | 2 | 2 | 287 | 1526 | sk__Eukaryota;Opisthokonta;k__Fungi;p__Glomeromycota;c__Glomeromycetes;o__Glomerales;f__Glomeraceae;g__Glomus;s__Glomus_sp._VeGlo10; |
| OTU_342 | 5 | 49 | 0 | 0 | 0 | 0 | 0 | 0 | 0 | 0 | 0 | 0 | 54 | sk__Eukaryota;Opisthokonta;k__Fungi;p__Glomeromycota;c__Glomeromycetes;o__Glomerales;f__Glomeraceae;g__Glomus;s__Glomus_sp._NBR_PP1; |
| OTU_166 | 21 | 4 | 6 | 0 | 8 | 0 | 0 | 0 | 0 | 74 | 54 | 263 | 430 | sk__Eukaryota;Opisthokonta;k__Fungi;p__Glomeromycota;c__Glomeromycetes;o__Glomerales;f__Glomeraceae;g__Glomus;s__Glomus_sp._C_3-10; |
| OTU_220 | 28 | 59 | 79 | 0 | 0 | 0 | 0 | 0 | 0 | 0 | 0 | 0 | 166 | sk__Eukaryota;Opisthokonta;k__Fungi;p__Glomeromycota;c__Glomeromycetes;o__Glomerales;f__Glomeraceae;g__Glomus;s__Glomus_sp._NBR_PP1; |
| OTU_691 | 4 | 1 | 0 | 0 | 0 | 0 | 0 | 2 | 1 | 0 | 0 | 0 | 8 | sk__Eukaryota;Opisthokonta;k__Fungi;p__Glomeromycota;c__Glomeromycetes;o__Glomerales;f__Glomeraceae;g__Septoglomus;s__Septoglomus_viscosum; |
| OTU_139 | 11 | 40 | 35 | 0 | 0 | 0 | 414 | 455 | 325 | 122 | 381 | 95 | 1878 | sk__Eukaryota;Opisthokonta;k__Fungi;p__Glomeromycota;c__Glomeromycetes;o__Glomerales;f__Glomeraceae;g__Septoglomus;s__Septoglomus_viscosum; |
| OTU_83 | 1 | 1 | 27 | 0 | 0 | 0 | 423 | 308 | 361 | 0 | 1 | 34 | 1156 | sk__Eukaryota;Opisthokonta;k__Fungi;p__Glomeromycota;c__Glomeromycetes;o__Glomerales;f__Glomeraceae;g__Glomus;s__Glomus_sp._VeGloB; |
| OTU_146 | 3 | 0 | 0 | 0 | 1 | 0 | 76 | 89 | 0 | 43 | 0 | 0 | 212 | sk__Eukaryota;Opisthokonta;k__Fungi;p__Glomeromycota;c__Glomeromycetes;o__Glomerales;f__Glomeraceae;g__Glomus;s__Glomus_sp._Glo9; |
| OTU_723 | 4 | 0 | 0 | 0 | 0 | 0 | 32 | 53 | 38 | 60 | 60 | 34 | 281 | sk__Eukaryota;Opisthokonta;k__Fungi;p__Glomeromycota;c__Glomeromycetes;o__Glomerales;f__Glomeraceae;g__Glomus;s__Glomus_sp._0904-1; |
| OTU_158 | 2 | 0 | 203 | 61 | 176 | 39 | 1 | 0 | 47 | 1 | 0 | 0 | 530 | sk__Eukaryota;Opisthokonta;k__Fungi;p__Glomeromycota;c__Glomeromycetes;o__Glomerales;f__Glomeraceae;g__Glomus;s__Glomus_indicum; |
| OTU_586 | 3 | 3 | 4 | 1 | 0 | 0 | 308 | 324 | 225 | 4 | 3 | 2 | 877 | sk__Eukaryota;Opisthokonta;k__Fungi;p__Glomeromycota;c__Glomeromycetes;o__Glomerales;f__Glomeraceae;g__Glomus;s__Glomus_sp._MO-G6; |
| OTU_92 | 1 | 15 | 0 | 54 | 0 | 0 | 285 | 158 | 73 | 0 | 0 | 0 | 586 | sk__Eukaryota;Opisthokonta;k__Fungi;p__Glomeromycota;c__Glomeromycetes;o__Diversisporales;f__Diversisporaceae;g__Diversispora;s__Diversispora_sp._S-MA-3; |
| OTU_101 | 1 | 3 | 0 | 0 | 1 | 0 | 2 | 160 | 0 | 124 | 253 | 32 | 576 | sk__Eukaryota;Opisthokonta;k__Fungi;p__Glomeromycota;c__Glomeromycetes;o__Glomerales;f__Glomeraceae;g__Glomus;s__Glomus_sp._PSAMG4; |
| OTU_97 | 0 | 40 | 116 | 0 | 0 | 0 | 0 | 0 | 117 | 0 | 129 | 12 | 414 | sk__Eukaryota;Opisthokonta;k__Fungi;p__Glomeromycota;c__Glomeromycetes;o__Glomerales;f__Glomeraceae;g__Glomus;s__Glomus_sp._Glo3b; |
| OTU_93 | 0 | 182 | 257 | 177 | 1 | 3 | 0 | 1 | 0 | 0 | 1 | 0 | 622 | sk__Eukaryota;Opisthokonta;k__Fungi;p__Glomeromycota;c__Glomeromycetes;o__Glomerales;f__Glomeraceae;g__Rhizophagus;s__Rhizophagus_sp._GB7; |
| OTU_28 | 0 | 101 | 0 | 0 | 1 | 0 | 407 | 423 | 888 | 776 | 576 | 169 | 3341 | sk__Eukaryota;Opisthokonta;k__Fungi;p__Glomeromycota;c__Glomeromycetes;o__Glomerales;f__Glomeraceae;g__Glomus;s__Glomus_sp._Glo9; |
| OTU_221 | 0 | 38 | 58 | 0 | 0 | 0 | 0 | 0 | 0 | 0 | 55 | 2 | 153 | sk__Eukaryota;Opisthokonta;k__Fungi;p__Glomeromycota;c__Glomeromycetes;o__Glomerales;f__Glomeraceae;g__Septoglomus;s__Septoglomus_viscosum; |
| OTU_348 | 0 | 110 | 1 | 0 | 1 | 0 | 0 | 0 | 0 | 0 | 0 | 0 | 112 | sk__Eukaryota;Opisthokonta;k__Fungi;p__Glomeromycota;c__Glomeromycetes;o__Glomerales;f__Glomeraceae;g__Glomus;s__Glomus_sp._C_3-10; |
| OTU_102 | 0 | 39 | 0 | 1 | 0 | 4 | 426 | 723 | 410 | 476 | 818 | 1082 | 3979 | sk__Eukaryota;Opisthokonta;k__Fungi;p__Glomeromycota;unclassified_Glomeromycota;s__Glomeromycota_sp._MIB_8856; |
| OTU_332 | 0 | 31 | 7 | 0 | 1 | 0 | 0 | 0 | 0 | 0 | 0 | 0 | 39 | sk__Eukaryota;Opisthokonta;k__Fungi;p__Glomeromycota;c__Glomeromycetes;o__Glomerales;f__Glomeraceae;g__Septoglomus;s__Septoglomus_viscosum; |
| OTU_415 | 0 | 2 | 0 | 0 | 0 | 8 | 0 | 0 | 13 | 0 | 0 | 0 | 23 | sk__Eukaryota;Opisthokonta;k__Fungi;p__Glomeromycota;c__Glomeromycetes;o__Glomerales;f__Glomeraceae;g__Glomus;s__Glomus_sp._MS; |
| OTU_145 | 0 | 16 | 42 | 0 | 0 | 0 | 38 | 181 | 52 | 0 | 0 | 0 | 329 | sk__Eukaryota;Opisthokonta;k__Fungi;p__Glomeromycota;c__Glomeromycetes;o__Glomerales;f__Glomeraceae;g__Glomus;s__Glomus_sp._Glo38; |
| OTU_173 | 0 | 11 | 37 | 0 | 2 | 6 | 104 | 66 | 10 | 0 | 0 | 0 | 236 | sk__Eukaryota;Opisthokonta;k__Fungi;p__Glomeromycota;c__Glomeromycetes;o__Glomerales;f__Glomeraceae;g__Glomus;s__Glomus_sp._MO-G8; |
| OTU_325 | 0 | 1 | 0 | 6 | 42 | 0 | 0 | 0 | 0 | 0 | 0 | 0 | 49 | sk__Eukaryota;Opisthokonta;k__Fungi;p__Glomeromycota;c__Glomeromycetes;o__Glomerales;f__Glomeraceae;g__Rhizophagus;s__Rhizophagus_intraradices; |
| OTU_715 | 0 | 1 | 0 | 36 | 0 | 0 | 0 | 0 | 0 | 0 | 0 | 0 | 37 | sk__Eukaryota;Opisthokonta;k__Fungi;p__Glomeromycota;c__Glomeromycetes;o__Glomerales;f__Glomeraceae;g__Glomus;s__Glomus_sp._NBR_PP1; |
| OTU_410 | 0 | 8 | 25 | 0 | 0 | 0 | 0 | 0 | 0 | 0 | 0 | 0 | 33 | sk__Eukaryota;Opisthokonta;k__Fungi;p__Glomeromycota;c__Glomeromycetes;o__Glomerales;f__Glomeraceae;g__Glomus;s__Glomus_sp._C_3-10; |
| OTU_60 | 0 | 1 | 0 | 0 | 1 | 0 | 216 | 215 | 291 | 0 | 0 | 0 | 724 | sk__Eukaryota;Opisthokonta;k__Fungi;p__Glomeromycota;c__Glomeromycetes;o__Glomerales;f__Glomeraceae;g__Glomus;s__Glomus_sp._Glo40; |
| OTU_285 | 0 | 1 | 0 | 56 | 0 | 1 | 0 | 0 | 0 | 0 | 0 | 0 | 58 | sk__Eukaryota;Opisthokonta;k__Fungi;p__Glomeromycota;c__Glomeromycetes;o__Glomerales;f__Glomeraceae;g__Glomus;s__Glomus_sp._MS; |
| OTU_432 | 0 | 0 | 13 | 4 | 0 | 2 | 0 | 0 | 0 | 0 | 0 | 0 | 19 | sk__Eukaryota;Opisthokonta;k__Fungi;p__Glomeromycota;c__Glomeromycetes;o__Archaeosporales;f__Ambisporaceae;g__Ambispora;s__Ambispora_leptoticha; |
| OTU_86 | 0 | 0 | 82 | 246 | 203 | 167 | 0 | 0 | 33 | 0 | 0 | 0 | 731 | sk__Eukaryota;Opisthokonta;k__Fungi;p__Glomeromycota;c__Glomeromycetes;o__Glomerales;f__Glomeraceae;g__Septoglomus;s__Septoglomus_viscosum; |
| OTU_658 | 0 | 0 | 8 | 0 | 0 | 0 | 0 | 0 | 0 | 0 | 0 | 0 | 8 | sk__Eukaryota;Opisthokonta;k__Fungi;p__Glomeromycota;c__Glomeromycetes;o__Glomerales;f__Glomeraceae;g__Glomus;s__Glomus_sp._MS; |
| OTU_772 | 0 | 0 | 0 | 565 | 35 | 161 | 2 | 0 | 0 | 0 | 0 | 0 | 763 | sk__Eukaryota;Opisthokonta;k__Fungi;p__Glomeromycota;unclassified_Glomeromycota;s__Glomeromycota_sp._WR856-B; |
| OTU_318 | 0 | 0 | 0 | 47 | 0 | 0 | 0 | 0 | 0 | 0 | 0 | 0 | 47 | sk__Eukaryota;Opisthokonta;k__Fungi;p__Glomeromycota;unclassified_Glomeromycota;s__Glomeromycota_sp._MIB_8386; |
| OTU_296 | 0 | 0 | 0 | 21 | 0 | 36 | 0 | 2 | 1 | 0 | 0 | 0 | 60 | sk__Eukaryota;Opisthokonta;k__Fungi;p__Glomeromycota;c__Glomeromycetes;o__Glomerales;f__Glomeraceae;g__Rhizophagus;s__Rhizophagus_iranicus; |
| OTU_246 | 0 | 0 | 0 | 46 | 56 | 81 | 0 | 0 | 66 | 0 | 0 | 0 | 249 | sk__Eukaryota;Opisthokonta;k__Fungi;p__Glomeromycota;c__Glomeromycetes;o__Glomerales;f__Glomeraceae;g__Glomus;s__Glomus_sp._MS; |
| OTU_237 | 0 | 0 | 0 | 64 | 1 | 53 | 0 | 0 | 0 | 0 | 0 | 0 | 118 | sk__Eukaryota;Opisthokonta;k__Fungi;p__Glomeromycota;c__Glomeromycetes;o__Glomerales;f__Glomeraceae;g__Glomus;s__Glomus_sp._NBR_PP1; |
| OTU_549 | 0 | 0 | 0 | 0 | 3 | 9 | 0 | 0 | 0 | 0 | 0 | 0 | 12 | sk__Eukaryota;Opisthokonta;k__Fungi;p__Glomeromycota;c__Glomeromycetes;o__Paraglomerales;f__Paraglomeraceae;g__Paraglomus;s__Paraglomus_sp._BSTA-3; |
| OTU_51 | 0 | 0 | 0 | 0 | 1 | 0 | 939 | 1229 | 597 | 0 | 0 | 0 | 2766 | sk__Eukaryota;Opisthokonta;k__Fungi;p__Glomeromycota;c__Glomeromycetes;o__Glomerales;f__Glomeraceae;g__Glomus;s__Glomus_sp._Glo38; |
| OTU_271 | 0 | 0 | 0 | 0 | 0 | 6 | 85 | 0 | 0 | 0 | 0 | 0 | 91 | sk__Eukaryota;Opisthokonta;k__Fungi;p__Glomeromycota;unclassified_Glomeromycota;s__Glomeromycota_sp._MIB_8875; |
| OTU_769 | 0 | 0 | 0 | 0 | 0 | 2 | 0 | 0 | 0 | 0 | 0 | 0 | 2 | sk__Eukaryota;Opisthokonta;k__Fungi;p__Glomeromycota;c__Glomeromycetes;o__Paraglomerales;f__Paraglomeraceae;g__Paraglomus;s__Paraglomus_sp._BSTA-3; |
| OTU_57 | 0 | 0 | 0 | 0 | 0 | 0 | 476 | 486 | 378 | 37 | 37 | 108 | 1522 | sk__Eukaryota;Opisthokonta;k__Fungi;p__Glomeromycota;c__Glomeromycetes;o__Glomerales;f__Glomeraceae;g__Glomus;s__Glomus_sp._Glo50; |
| OTU_255 | 0 | 0 | 0 | 0 | 0 | 0 | 112 | 0 | 0 | 0 | 0 | 0 | 112 | sk__Eukaryota;Opisthokonta;k__Fungi;p__Glomeromycota;c__Glomeromycetes;o__Diversisporales;f__Gigasporaceae;g__Gigaspora;s__Gigaspora_margarita; |
| OTU_679 | 0 | 0 | 0 | 0 | 0 | 0 | 14 | 6 | 4 | 12 | 5 | 20 | 61 | sk__Eukaryota;Opisthokonta;k__Fungi;p__Glomeromycota;c__Glomeromycetes;o__Glomerales;f__Glomeraceae;g__Glomus;s__Glomus_sp._Glo25; |
| OTU_234 | 0 | 0 | 0 | 0 | 0 | 0 | 55 | 0 | 0 | 55 | 29 | 7 | 146 | sk__Eukaryota;Opisthokonta;k__Fungi;p__Glomeromycota;c__Glomeromycetes;o__Glomerales;f__Glomeraceae;unclassified_Glomeraceae;s__Glomales_sp._other1; |
| OTU_500 | 0 | 0 | 0 | 0 | 0 | 0 | 16 | 0 | 0 | 0 | 0 | 0 | 16 | sk__Eukaryota;Opisthokonta;k__Fungi;p__Glomeromycota;c__Glomeromycetes;o__Glomerales;f__Glomeraceae;g__Glomus;s__Glomus_sp._SS06-4; |
| OTU_343 | 0 | 0 | 0 | 0 | 0 | 0 | 52 | 22 | 30 | 0 | 0 | 0 | 104 | sk__Eukaryota;Opisthokonta;k__Fungi;p__Glomeromycota;c__Glomeromycetes;o__Glomerales;f__Glomeraceae;g__Glomus;s__Glomus_sp._Glo50; |
| OTU_538 | 0 | 0 | 0 | 0 | 0 | 0 | 13 | 0 | 0 | 0 | 0 | 0 | 13 | sk__Eukaryota;Opisthokonta;k__Fungi;p__Glomeromycota;c__Glomeromycetes;o__Glomerales;f__Glomeraceae;g__Rhizophagus;s__Rhizophagus_intraradices; |
| OTU_430 | 0 | 0 | 0 | 0 | 0 | 0 | 30 | 0 | 0 | 0 | 0 | 0 | 30 | sk__Eukaryota;Opisthokonta;k__Fungi;p__Glomeromycota;c__Glomeromycetes;o__Glomerales;f__Glomeraceae;g__Glomus;s__Glomus_sp._Glo40; |
| OTU_238 | 0 | 0 | 0 | 0 | 0 | 0 | 6 | 0 | 0 | 64 | 6 | 17 | 93 | sk__Eukaryota;Opisthokonta;k__Fungi;p__Glomeromycota;c__Glomeromycetes;o__Paraglomerales;f__Paraglomeraceae;g__Paraglomus;s__Paraglomus_sp._BSTA-3; |
| OTU_258 | 0 | 0 | 0 | 0 | 0 | 0 | 0 | 83 | 50 | 0 | 0 | 0 | 133 | sk__Eukaryota;Opisthokonta;k__Fungi;p__Glomeromycota;c__Glomeromycetes;o__Glomerales;f__Glomeraceae;g__Glomus;s__Glomus_sp._MO-G14; |
| OTU_560 | 0 | 0 | 0 | 0 | 0 | 0 | 0 | 0 | 17 | 0 | 0 | 0 | 17 | sk__Eukaryota;Opisthokonta;k__Fungi;p__Glomeromycota;c__Glomeromycetes;o__Diversisporales;f__Diversisporaceae;g__Redeckera;s__Redeckera_fulvum; |
| OTU_441 | 0 | 0 | 0 | 0 | 0 | 0 | 0 | 0 | 0 | 31 | 1 | 0 | 32 | sk__Eukaryota;Opisthokonta;k__Fungi;p__Glomeromycota;unclassified_Glomeromycota;s__Glomeromycota_sp._WR331.b-C; |
| OTU_742 | 0 | 0 | 0 | 0 | 0 | 0 | 0 | 0 | 0 | 33 | 3 | 0 | 36 | sk__Eukaryota;Opisthokonta;k__Fungi;p__Glomeromycota;c__Glomeromycetes;o__Glomerales;f__Glomeraceae;g__Glomus;s__Glomus_sp._BSLA-7; |
| OTU_519 | 0 | 0 | 0 | 0 | 0 | 0 | 0 | 0 | 0 | 14 | 0 | 0 | 14 | sk__Eukaryota;Opisthokonta;k__Fungi;p__Glomeromycota;c__Glomeromycetes;o__Diversisporales;f__Diversisporaceae;g__Diversispora;s__Diversispora_sp._MAY-2012; |
| OTU_194 | 0 | 0 | 0 | 0 | 0 | 0 | 0 | 0 | 0 | 0 | 39 | 176 | 215 | sk__Eukaryota;Opisthokonta;k__Fungi;p__Glomeromycota;c__Glomeromycetes;o__Glomerales;f__Glomeraceae;g__Glomus;s__Glomus_sp._PP; |
| OTU_273 | 0 | 0 | 0 | 0 | 0 | 0 | 0 | 0 | 0 | 0 | 93 | 0 | 93 | sk__Eukaryota;Opisthokonta;k__Fungi;p__Glomeromycota;unclassified_Glomeromycota;s__Glomeromycota_sp._AB-2014; |
| OTU_578 | 0 | 0 | 0 | 0 | 0 | 0 | 0 | 0 | 0 | 0 | 9 | 0 | 9 | sk__Eukaryota;Opisthokonta;k__Fungi;p__Glomeromycota;c__Glomeromycetes;o__Archaeosporales;f__Ambisporaceae;g__Ambispora;s__Ambispora_sp._JP-2009-1; |
| OTU_741 | 0 | 0 | 0 | 0 | 0 | 0 | 0 | 0 | 0 | 0 | 31 | 98 | 129 | sk__Eukaryota;Opisthokonta;k__Fungi;p__Glomeromycota;c__Glomeromycetes;o__Glomerales;f__Glomeraceae;g__Glomus;s__Glomus_sp._C_3-10; |
