# Supplementary figures and images for: Species diversity and drivers of arbuscular mycorrhizal fungal communities in a semi-arid mountain in China
Source: PeerJ. 2017 Dec 8;5:e4155. doi: 10.7717/peerj.4155 (PMC5724403; doi:10.7717/peerj.4155)

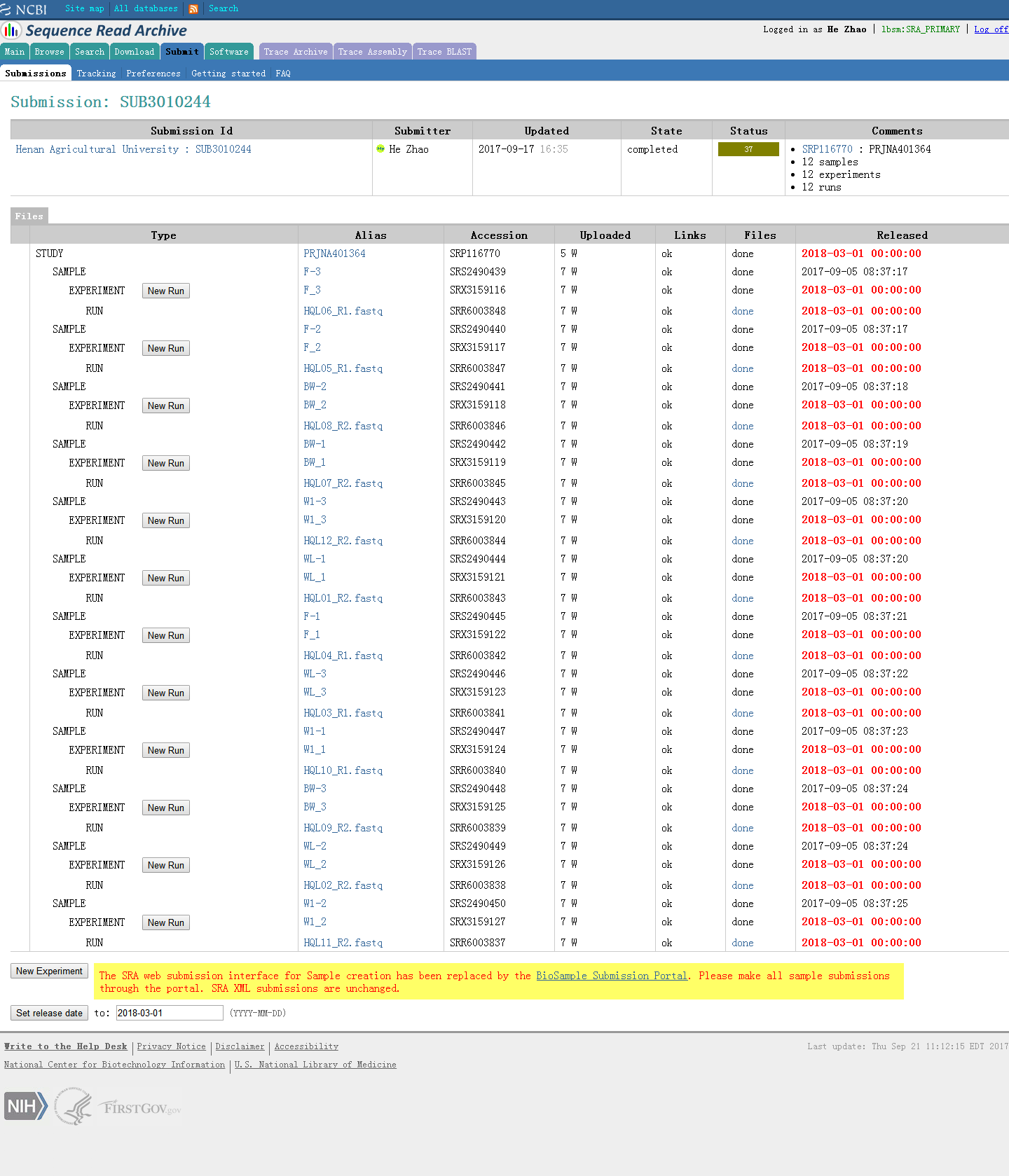

Supplement: Supplemental Information 13 [file peerj-05-4155-s016.png]
